# A New Adaptive GCC Method and Its Application to Slug Flow Velocity Measurement in Small Channels

**DOI:** 10.3390/s22093160

**Published:** 2022-04-20

**Authors:** Hua Xia, Junchao Huang, Haifeng Ji, Baoliang Wang, Zhiyao Huang

**Affiliations:** State Key Laboratory of Industrial Control Technology, College of Control Science and Engineering, Zhejiang University, Hangzhou 310027, China; xhua@zju.edu.cn (H.X.); hfji@zju.edu.cn (H.J.); wangbl@zju.edu.cn (B.W.); zy_huang@zju.edu.cn (Z.H.)

**Keywords:** adaptive, cross-correlation, velocity measurement, slug flow, small channels

## Abstract

In this work, an adaptive generalized cross-correlation (AGCC) method is proposed that focuses on the problem of the conventional cross-correlation method not effectively realizing the time delay estimation of signals with strong periodicity. With the proposed method, the periodicity of signals is judged and the center frequencies of the strongly periodical components are determined through the spectral analysis of the input signals. Band-stop filters that are used to suppress the strongly periodical components are designed and the mutual power spectral density of the input signals that is processed by the band-stop filters is calculated. Then, the cross-correlation function that is processed is the inverse Fourier transform of the mutual power spectral density. Finally, the time delay is estimated by seeking the peak position of the processed cross-correlation function. Simulation experiments and practical velocity measurement experiments were carried out to verify the effectiveness of the proposed AGCC method. The experimental results showed that the new AGCC method could effectively realize the time delay estimation of signals with strong periodicity. In the simulation experiments, the new method could realize the effective time delay estimation of signals with strong periodicity when the energy ratio of the strongly periodical component to the aperiodic component was under 150. Meanwhile, the cross-correlation method and other generalized cross-correlation methods fail in time delay estimation when the energy ratio is higher than 30. In the practical experiments, the velocity measurement of slug flow with strong periodicity was implemented in small channels with inner diameters of 2.0 mm, 2.5 mm and 3.0 mm. With the proposed method, the relative errors of the velocity measurement were less than 4.50%.

## 1. Introduction

The cross-correlation method is widely used in many fields for time delay estimation, such as for the flow rate/velocity measurement of fluids [1,2,3,4,5,6,7,8,9]. Wang et al. utilized the cross-correlation method to calculate the particle velocities between the electrodes from two planes at different positions and to establish the particle velocity distributions in pipelines [5]. Bai et al. designed a distributed four-sector conductance cross-correlation sensor to realize the measurement of oil–water flow velocity in a pipe with a 20 mm inner diameter [6]. To measure the differences in times of arrival in the sound source localization field [10,11,12,13,14,15], Padois el al. designed and optimized microphone array geometry and used the generalized cross-correlation method to realize the localization of acoustic sources [12,13]. Manuel Vera-Diaz et al. combined a convolutional deep neural network and the generalized cross-correlation method to create a 3D acoustic map that could recover source positions [15]. In the time delay estimation of biological responses [16,17], Hong et al. designed a signal quality index that was based on the cross-correlation result between the observed signals from a multimodal biosignal chair and the noise-free signals; hence, they could assess the practical use of the chair for smart healthcare [16]. Wang et al. applied the cross-correlation method to the auditory brainstem response test and found a good match between their proposed method and the human readouts [17]. Nevertheless, in practical applications, the conventional cross-correlation methods are difficult to use effectively for the time delay estimation of signals with strong periodicity [1,2] because it is difficult to find a peak in the cross-correlation function of signals with strong periodicity. Additionally, the maximum position of the cross-correlation function does not represent the practical time delay [1,2]. There is a lack of research on the time delay estimation of signals with strong periodicity and it is essential to discover an effective method to deal with this problem and fill that research gap.

Recently, gas–liquid two-phase flows in small channels have received more attention from researchers and engineers due to their high mass, heat transfer performance and compact structure [18,19,20]. Slug flow is one of the most common flow patterns of gas–liquid two-phase flows in small channels [21,22,23]. Haase et al. indicated the potential of slug flow in intensity reaction processes and provided an overview of the hydrodynamics and mass transfer characteristics of slug flow [19]. Therefore, it is important to realize the measurement of slug flow velocity in small channels both for academic research and industrial applications [18,19,20,21,22,23]. However, the conventional cross-correlation method is not suitable for the measurement of slug flow velocity in small channels [18,19,20,21,22,23] because the conventional cross-correlation methods were developed for the measurement of gas–liquid two-phase flow velocity in normal scale pipelines and because the periodicity of slug flow is not obvious or strong. While with the decrease in the inner diameters of small channels, the measurement signals in the small channels are likely to appear to have strong periodicity. When the conventional method is applied to measurement of slug flow velocity in small channels, intolerable errors may occur because of the strong periodicity [1,2]. Therefore, it is critical to develop a new cross-correlation method that can effectively process signals with strong periodicity, estimate time delay and hence, implement the measurement of slug flow velocity with strong periodicity.

**Figure 1:**
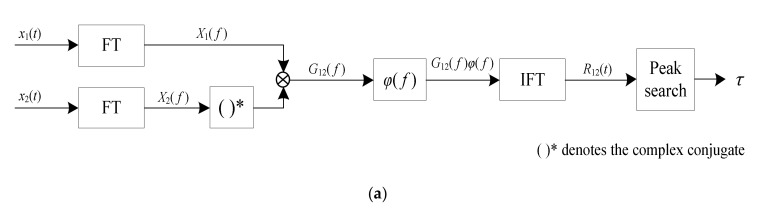

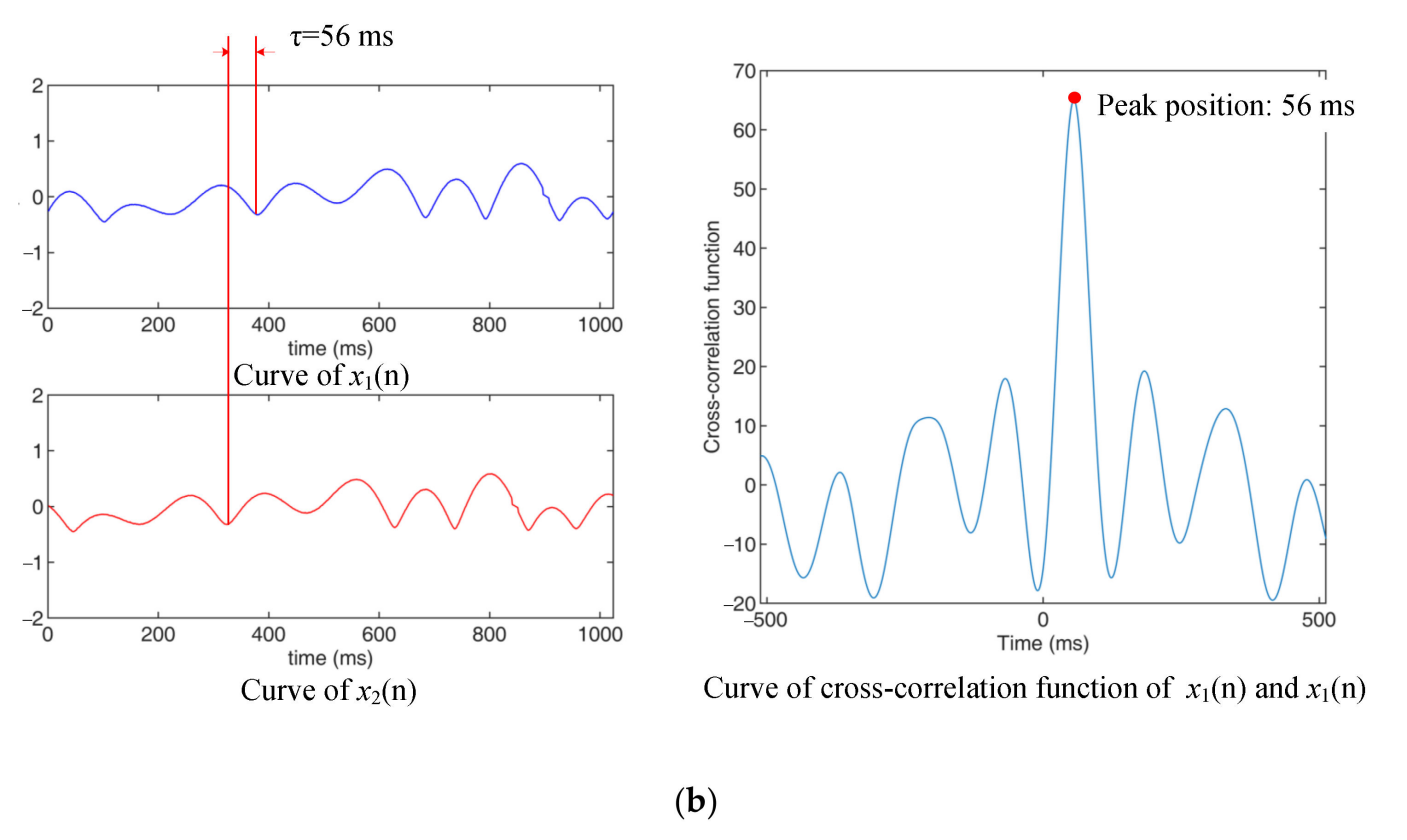
Time delay estimation principle of the GCC method: (**a**) flowchart of the GCC method; (**b**) a typical example of time delay estimation using the cross-correlation method.

The generalized cross-correlation (GCC) method provides a possible approach. The generalized cross-correlation (GCC) method was developed by expanding the cross-correlation method [24,25,26,27]. Unlike the basic cross-correlation method, the generalized cross-correlation method can sharpen the peak of the cross-correlation function and improve time delay estimation performance by introducing a weight function when implementing the time delay estimation [24,25,26,27]. Figure 1 shows the time delay estimation principle of the GCC method. Figure 1a is a flowchart of the GCC method. Figure 1b shows a typical example of time delay estimation using the cross-correlation method.

As shown in Figure 1a, $x_{1}\left(t\right)$ and $x_{2}\left(t\right)$ are the two input signals. With the GCC method, the spectra of the two input signals $X_{1}\left(f\right)$ and $X_{2}\left(f\right)$ are obtained using the Fourier transform (FT) and then the mutual power spectral density of the two input signals $G_{12}\left(f\right)$ can be obtained. Then, a weight function $\varphi \left(f\right)$ is introduced and multiplied by the mutual power spectral density and hence, obtains the processed mutual power spectral density $G_{12}\left(f\right)\;\varphi \left(f\right)$. The cross-correlation function $R_{12}\left(t\right)$ is obtained using the mutual power spectral density that was processed with the inverse Fourier transform (IFT). The time delay is obtained from the position of the peak of the cross-correlation function, as shown in Figure 1b. The weight function $\varphi \left(f\right)$ is the key point of the GCC method. Some weight functions have been proposed, such as the Roth [24,26], PHAT [24,27], SCOT [24,27], etc. Unfortunately, the conventional GCC methods with different weight functions $\varphi \left(f\right)$ were designed to sharpen the peak of the cross-correlation function and hence, improve the time delay estimation performance [24,25,26,27]. These GCC methods hardly take the influence of the strong periodicity of measurement signals on time delay estimation into consideration [24,25,26,27]. Therefore, in order to effectively realize the time delay estimation of signals with strong periodicity, it is necessary to develop a new GCC method that can properly process signals with strong periodicity.

In this paper, a new adaptive generalized cross-correlation (AGCC) method is proposed for the time delay estimation of signals with strong periodicity. Two adaptive band-stop filters were introduced into the new adaptive generalized cross-correlation (AGCC) method. The signals with strong periodicity were processed by the two adaptive band-stop filters and the time delay estimation of signals with strong periodicity was implemented. Numerical simulations were carried out to test the performance of the proposed AGCC method. The measurement of slug flow velocity in small channels is then introduced as an example of the practical application of the proposed method and further verifies the effectiveness of the proposed AGCC method.

## 2. New Adaptive Generalized Cross-Correlation (AGCC) Method

### 2.1. Principles of the AGCC Method

**Figure 2:**
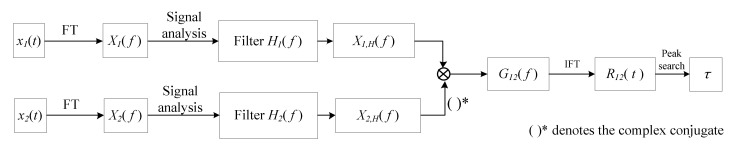
Flowchart of the new adaptive GCC method.

Figure 2 shows a flowchart of the new adaptive generalized cross-correlation (AGCC) method.

As shown in Figure 2, $x_{1}\left(t\right)$ and $x_{2}\left(t\right)$ are the two input signals. The spectra of the two input signals $X_{1}\left(f\right)$ and $X_{2}\left(f\right)$ are obtained by the Fourier transform (FT):

(1) $$X_{1}\left(f\right)=\int_{-\infty }^{\infty }x_{1}\left(t\right)e^{-j2\pi ft}dt$$

(2) $$X_{2}\left(f\right)=\int_{-\infty }^{\infty }x_{2}\left(t\right)e^{-j2\pi ft}dt$$

where, $X_{1}\left(f\right)$ is the spectrum of $x_{1}\left(t\right)$ and $X_{2}\left(f\right)$ is the spectrum of $x_{2}\left(t\right)$. The spectra of the input signals are analyzed and two filters $H_{1}\left(f\right)$ and $H_{2}\left(f\right)$ are adaptively designed according to the signal analysis results. The strong periodicity components of $X_{1}\left(f\right)$ and $X_{2}\left(f\right)$ are suppressed by the operation of the two filters. Two filtered signals $X_{1,H}\left(f\right)$ and $X_{2,H}\left(f\right)$ are then obtained:

(3) $$X_{1,H}\left(f\right)=X_{1}\left(f\right)H_{1}\left(f\right)$$

(4) $$X_{2,H}\left(f\right)=X_{2}\left(f\right)H_{2}\left(f\right)$$

The mutual power spectral density of the two filtered signals is $G_{12}\left(f\right)$:

(5) $$G_{12}\left(f\right)=X_{1,H}\left(f\right){\left(X_{2,H}\left(f\right)\right)}^{*}$$

where ${\left(\;\right)}^{*}$ denotes the complex conjugate. $G_{12}\left(f\right)$ is the Fourier transform of the cross-correlation function of the two filtered signals. Thus, the cross-correlation function $R_{12}\left(t\right)$ can be obtained when the inverse Fourier transform is applied to $G_{12}\left(f\right)$:

(6) $$R_{12}\left(t\right)=\int_{-\infty }^{\infty }G_{12}\left(f\right)e^{j2\pi ft}df$$

Finally, by searching for the peak position of $R_{12}\left(t\right)$, the time delay estimation is implemented and the time delay τ is the peak position of $R_{12}\left(t\right)$.

As mentioned above, the target of the conventional GCC methods is to sharpen the peak of the cross-correlation function, while the proposed AGCC method aims to overcome the influence of the strong periodicity of measurement signals on time delay estimation. By comparing a conventional GCC method (as shown in Figure 1a) and the proposed AGCC method, it was found that the main difference between the methods is that the conventional GCC method introduces a weight function $\varphi \left(f\right)$ to process the mutual power spectral density of the input signals, while the AGCC method introduces two filters that are adaptively designed according to the signal analysis to process the spectra of the input signals. Obviously, the design of $H_{1}\left(f\right)$ and $H_{2}\left(f\right)$ is important for effective time delay estimation. In addition, the filters need to be adaptive filters.

**Figure 3:**
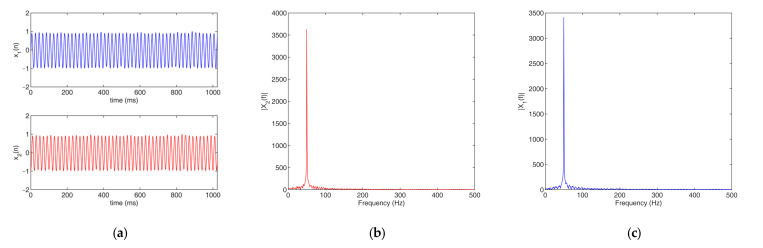
A typical example of signals with strong periodicity: (**a**) signals; (**b**) spectra of $x_{1}\left(t\right)$; (**c**) spectra of $x_{2}\left(t\right)$.

In the AGCC method, the two filters are adaptive band-stop filters. As there is only a time delay between the two input signals $x_{1}\left(t\right)$ and $x_{2}\left(t\right)$, the magnitude responses of their spectra are almost the same and the filters of $x_{1}\left(t\right)$ and $x_{2}\left(t\right)$ are the same. Therefore, in this work, we only needed to analyze either $x_{1}\left(t\right)$ or $x_{2}\left(t\right)$ to determine the center frequency of the stopband of the two filters $H_{1}\left(f\right)$ and $H_{2}\left(f\right)$. In addition, there is an obvious feature in the magnitude response of spectrum for a signal that contains strong periodicity components, i.e., a prominent and obvious peak can be found in the frequency that corresponds to the strong periodicity components. Figure 3 shows an example of this phenomenon.

To design the two adaptive band-stop filters ($H_{1}\left(f\right)$ and $H_{2}\left(f\right)$), the input signal $x\left(t\right)$ ($x_{1}\left(t\right)$ or $x_{2}\left(t\right)$) is processed using the Fourier transform. The spectrum of the signal $X\left(f\right)$ and its magnitude $\left|X\left(f\right)\right|$ are obtained. Then, the frequency position $f_{max}$ is found where the corresponding magnitude is the largest so that the largest magnitude $\left|X\left(f_{max}\right)\right|$ satisfies the following condition (Equation (7)):

(7) $$\left|X\left(f_{max}\right)\right|\geq \mu +k\sigma$$

where μ and σ are the mean value and the standard deviation of $\left|X\left(f\right)\right|$ and k is the periodic judgement coefficient. In this work, k was determined in advance according to previous experiments and later in this article, k was set as 3. This means that the periodicity component whose frequency is $f_{max}$ is too strong for the effective implementation of time delay estimation and needs to be suppressed. The center frequency of the stopband of the adaptive filters is $f_{max}$. Further, the stopband of the filter is:

(8) $$\left(1-\varepsilon \right)f_{max}<f<\left(1+\varepsilon \right)f_{max}$$

where ε is the filter range coefficient.

Thus, based on the above descriptions, the two corresponding adaptive band-stop filters $H_{1}\left(f\right)$ and $H_{2}\left(f\right)$ of $x_{1}\left(t\right)$ and $x_{2}\left(t\right)$ are:

(9) H1f=1f<1−εfmax 01−εfmax<f<1+εfmax1 f>1+εfmax H2f=1f<1−εfmax01−εfmax<f<1+εfmax1f>1−εfmax

**Figure 4:**
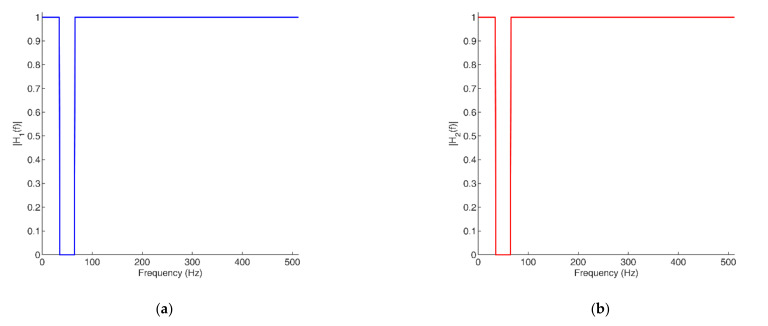
Magnitude responses of $H_{1}\left(f\right)$ and $H_{2}\left(f\right)$: (**a**) $H_{1}\left(f\right)$; (**b**) $H_{2}\left(f\right)$.

Figure 4 illustrates the magnitude responses of the band-stop filters $H_{1}\left(f\right)$ and $H_{2}\left(f\right)$ that were designed for the signals in Figure 3.

### 2.2. Digital Implementation of the AGCC Method

In practical applications, the information that is obtained is digital, i.e., the input signals ($x_{1}\left(t\right)$ and $x_{2}\left(t\right)$) are digital data. The Fourier transform (FT) and inverse Fourier transform (IFT) are realized by digital Fourier transform (DFT) and inverse digital Fourier transform (IDFT), respectively. The signal processing of time delay estimation can be mainly divided into the following six steps:

(1) To obtain the digital input sequences $x_{1}\left(n\Delta t\right)$ and $x_{2}\left(n\Delta t\right)$ by sampling the input signals ($x_{1}\left(t\right)$ and $x_{2}\left(t\right)$) with the A/D converters where Δt is the sampling time interval, n=0,1,2…,N−1 is the index of the sequences and N is the sampling number, the sampling duration T and the sampling frequency $f_{s}$ can be expressed as:
  (10) T=NΔt
  (11) $$f_{s}=\frac{1}{\Delta t};$$
(2) Then, obtain the spectra of the digital input sequences $X_{1}\left(f_{i}\right)$ and $X_{2}\left(f_{i}\right)$ by DFT:
  (12) $$X_{1}\left(f_{i}\right)=\overset{N-1}{\underset{n=0}{\sum }}x_{1}\left(n\Delta t\right)e^{-j2\pi f_{i}n\Delta t}$$
  (13) $$X_{2}\left(f_{i}\right)=\overset{N-1}{\underset{n=0}{\sum }}x_{2}\left(n\Delta t\right)e^{-j2\pi f_{i}n\Delta t}$$
  where $i=0,1,2,\ldots ,\frac{N}{2}$ is the index of the frequency and $f_{i}=\frac{i}{\;N\Delta t}$ is the frequency sequence of the spectra. In practical applications, the fast Fourier transform (FFT) algorithm is adopted to obtain of the spectra;
(3) Design the two adaptive band-stop filters ($H_{1}\left(f_{i}\right)$ and $H_{2}\left(f_{i}\right)$), i.e., determine the stopband of $H_{1}\left(f_{i}\right)$ and $H_{2}\left(f_{i}\right)$.

Using $X_{1}\left(f_{i}\right)$ as an example, the magnitude response $X_{1}\left(f\right)$ needs to be calculated, i.e., $\left|X_{1}\left(f_{i}\right)\right|$. Then, determine the frequency position of the largest magnitude $f_{max}$ and the largest magnitude $\left|X_{1}\left(f_{max}\right)\right|$. Judge whether the periodicity component is too strong and needs to be eliminated by $\left|X\left(f_{max}\right)\right|\geq \mu +k\sigma$. The mean value μ and the standard deviation σ are determined as:

(14) $$\mu =\frac{1}{N/2+1}\overset{\frac{N}{2}}{\underset{i=0}{\sum }}\;\left|X_{1}\left(f_{i}\right)\right|$$

(15) $$\sigma =\sqrt{\frac{1}{N/2}\overset{\frac{N}{2}}{\underset{i=0}{\sum }}\;{\left[\mu -\left|X_{1}\left(f_{i}\right)\right|\;\right]}^{2}}$$

The two band-stop filters $H_{1}\left(f\right)$ and $H_{2}\left(f\right)$ are designed as:

(16) H1fi=1fi<1−εfmax 01−εfmax<fi<1+εfmax1 fi>1+εfmax H2fi=1fi<1−εfmax01−εfmax<fi<1+εfmax1fi>1−εfmax

If the periodicity component is not strong, i.e., $\left|X\left(f_{max}\right)\right|\leq \mu +k\sigma$, that means that there is no obvious peak in the magnitude responses of spectrum of the input signals and the basic cross-correlation method can be implemented for effective time delay estimation. In this case, $H_{1}\left(f_{i}\right)=H_{2}\left(f_{i}\right)=1$;

(4) To obtain the spectra of the filtered signals $X_{1,H}\left(f_{i}\right)$ and $X_{2,H}\left(f_{i}\right)$:
  (17) $$X_{1,H}\left(f_{i}\right)=X_{1}\left(f_{i}\right)H_{1}\left(f_{i}\right)$$
  (18) $$X_{2,H}\left(f_{i}\right)=X_{2}\left(f_{i}\right)H_{2}\left(f_{i}\right)$$

To obtain the mutual power spectral density of the filtered signals $G_{12}\left(f_{i}\right)$:

(19) $$G_{12}\left(f_{i}\right)=X_{1,H}\left(f_{i}\right){\left(X_{2,H}\left(f_{i}\right)\right)}^{*};$$

(5) To obtain the cross-correlation function $R_{12}\left(m\Delta t\right)$ by IDFT (IFFT):
  (20) $$R_{12}\left(m\Delta t\right)=\sum_{i=-\frac{N}{2}+1}^{\frac{N}{2}}G_{12}\left(f_{i}\right)e^{j2\pi f_{i}m\Delta t}m=-\frac{N}{2}+1,\;\ldots -1,0,1,\ldots \frac{N}{2};$$
(6) Find the peak position of $R_{12}\left(m\Delta t\right)$ to determine the time delay of the two input signals.

**Figure 5:**
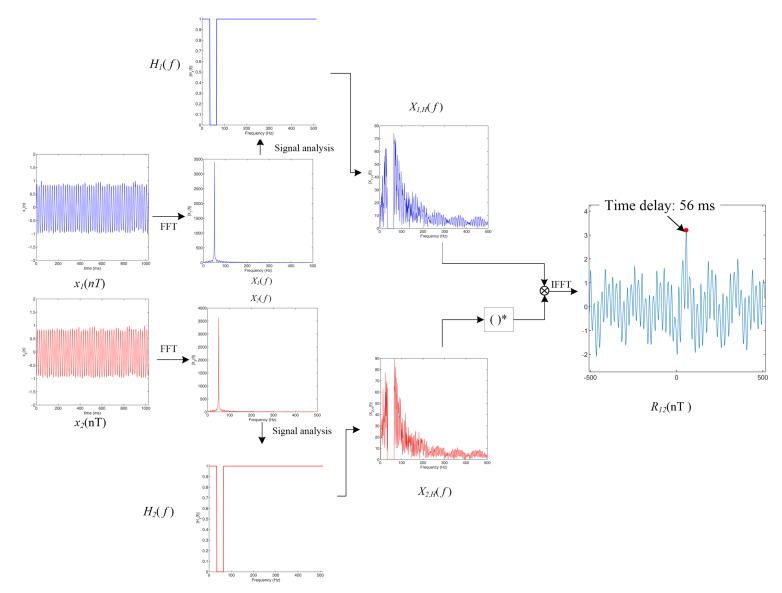
New adaptive GCC method for the signals in Figure 3 (the filter range coefficient ε = 0.05).

Figure 5 shows the process of using the new AGCC method for the time delay estimation of signals with strong periodicity (using the signal in Figure 3 as an example).

### 2.3. Modification for Practical Measurements

**Figure 6:**
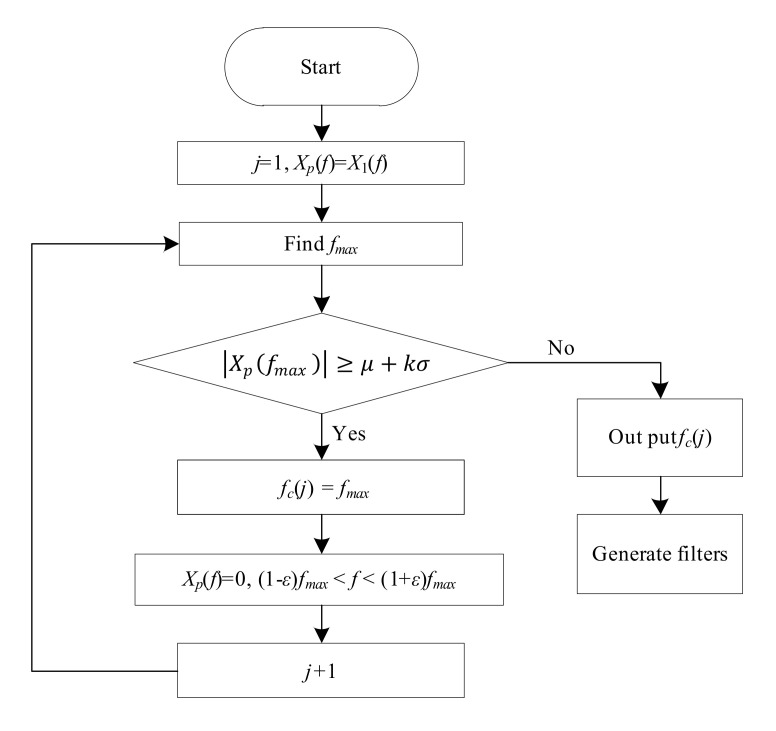
Signal analysis and the generation of band-stop filters in practical measurements.

It is necessary to indicate that in practical measurements, signals with strong periodicity usually have more than one strongly periodical component. In the case of a signal with strong periodicity having multiple strongly periodical components, the flowchart of the time delay estimation needs to be modified. Figure 6 shows the signal analysis and the generation of the band-stop filters in practical measurements.

According to Figure 6, the signal analysis process in practical measurements is as follows:

Step 1. Initialize the processed signal spectra $X_{p}\left(f\right)=X_{1}\left(f\right)$, set the iteration number j=1 and initialize the periodical center frequency set $f_{c}\left(j\right)$ as an empty set;
Step 2. Calculate the magnitude responses $\left|X_{p}\left(f\right)\right|$. Then, find the frequency position $f_{max}$ where the corresponding magnitude is the largest;
Step 3. Judge the periodical intensity according to $\left|X_{p}\left(f_{max}\right)\right|\geq \mu +k\sigma$. When $\left|X_{p}\left(f_{max}\right)\right|\geq \mu +k\sigma$, record $f_{c}\left(j\right)=f_{max}$ and go to Step 4. When $\left|X_{p}\left(f_{max}\right)\right|<\mu +k\sigma$, output $f_{c}\left(i\right)$ and go to Step 5;
Step 4. Set the frequency range $\left(1-\varepsilon \right)f_{max}<f<\left(1+\varepsilon \right)f_{max}$ of $X_{p}\left(f\right)=0$ and set the iteration number j=j+1. Then, return to Step 2;
Step 5. Generate the two band-stop filters $H_{1}\left(f\right)$ and $H_{2}\left(f\right)$:
  (21) $$\begin{array}{c} H_{1}\left(f\right)=H_{2}\left(f\right)=\left\{\begin{array}{cc} 1 & f<\left(1-\varepsilon \right)f_{c}\left(j\right)\;\\ 0 & \left(1-\varepsilon \right)f_{c}\left(j\right)<f<\left(1+\varepsilon \right)f_{c}\left(j\right)\;\\ 1 & \;f>\left(1+\varepsilon \right)f_{c}\left(j\right)\; \end{array}\right. \end{array}$$

With the obtained band-stop filters, the remaining steps are the same as the AGCC method that was introduced above, i.e., obtain the spectra of the filtered signals (Equations (3) and (4)), obtain the mutual power spectral density of the filtered signals (Equation (5)), obtain the cross-correlation function (Equations (6)) and find the peak position of the cross-correlation function to determine the time delay of the two input signals.

## 3. Experimental Results

### 3.1. Simulation Experiments

Simulation experiments were carried out to test the effectiveness of the proposed method for signals with strong periodicity. In the simulation experiments, test signals with different strongly periodical components were generated. Then, the time delay estimation results of those test signals from the different cross-correlation methods (CC, GCC with Roth, GCC with PHAT and AGCC) were compared and analyzed. 

The original test signal $F\left(n\right)$ was:

(22) $$F\left(n\right)=P\left(n\right)+E\left(n\right)$$

where $P\left(n\right)$ is the strongly periodical component and $E\left(n\right)$ is the aperiodic fluctuant component:

(23) $$P\left(n\right)=A_{m}\sin\left(2\pi f_{c}n\Delta t\right)$$

where $A_{m}$ is the amplitude, $f_{c}$ is the periodical center frequency and Δt is the sample interval of the signal. $E\left(n\right)$ is the aperiodic fluctuant component that was generated randomly. Thus, by adjusting the $A_{m}$, the original test signal could show different degrees of periodicity. The two input signals $x_{1}\left(n\right)$ and $x_{2}\left(n\right)$ were two clips that were taken from the $F\left(n\right)$ and the time difference between $x_{1}\left(n\right)$ and $x_{2}\left(n\right)$ was the reference time delay. The ratio of the signal energy RE was used as the strong periodicity index of the degrees of periodicity:

(24) $$RE=\frac{\sum_{n=1}^{N}{\left[P\left(n\right)\right]}^{2}}{\sum_{n=1}^{N}{\left[E\left(n\right)\right]}^{2}}$$

where N is the length of the original test signal.

Table 1 lists the experimental results of the simulation experiments.

**Figure 7:**
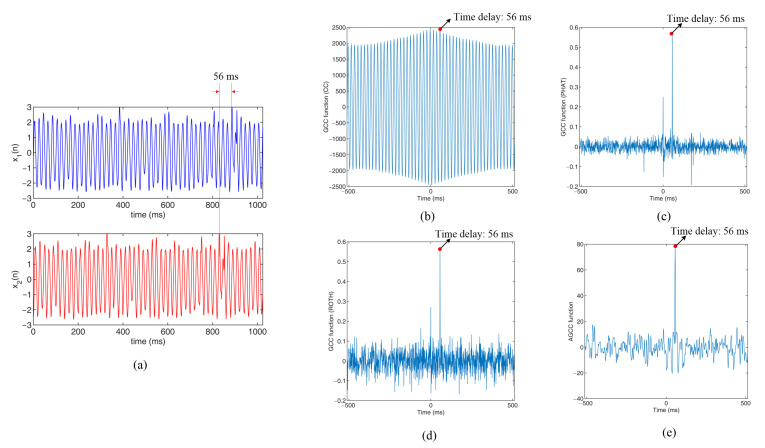
Results of the simulation experiments with $A_{m}=30$ and RE=7.6: (**a**) original signals; (**b**) cross-correlation functions obtained by CC; (**c**) cross-correlation functions obtained by GCC with Roth; (**d**) cross-correlation functions obtained by GCC with PHAT; (**e**) cross-correlation functions obtained by AGCC.

To better compare the characteristics and performances of the different cross-correlation methods, two groups of simulation experiments ($A_{m}=30$ and RE=7.6; $A_{m}=100$ and RE=85.6) were used as examples to show the cross-correlation functions that were obtained from the different cross-correlation methods. Figure 7 shows the results of the simulation experiments with $A_{m}=30$ and RE=7.6. Figure 7a shows the original signals and Figure 7b–e are the cross-correlation functions that were obtained by the CC (conventional cross-correlation) method, the GCC method with Roth, the GCC method with PHAT and the new AGCC method, respectively.

In Figure 7a, it can be seen that the input signals had some random fluctuations and the time delay of the two signals can be easily observed by the naked eye. Meanwhile, due to the existence of the strongly periodical component $A_{m}=30$, the signals had obvious periodicity, which led to the strong periodicity of the cross-correlation functions that were obtained by CC, as shown in Figure 7b. In Figure 7b, although peak position of the cross-correlation function τ=56 ms was the correct time delay, the values of the cross-correlation functions were very close to the values of other peak positions, such as τ=−4 ms, 16 ms and 36 ms. In Figure 7c,d, the peaks of the GCC functions are apparent and their positions were correct. However, there was a second peak at the position τ=0 ms and the experiment results showed that the second peak increased with the increase in the degree of periodicity. In Figure 7e, it can be seen that the cross-correlation function could realize effective time delay estimation.

**Figure 8:**
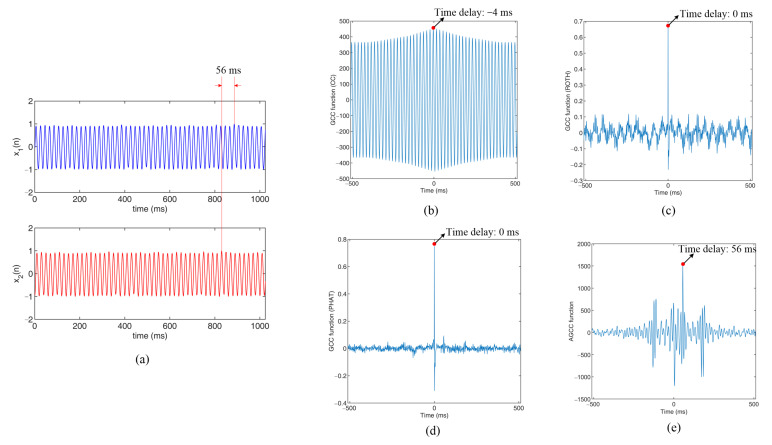
Results of the simulation experiments with $A_{m}=100$ and RE=85.6: (**a**) original signals; (**b**) cross-correlation functions obtained by CC; (**c**) cross-correlation functions obtained by GCC with Roth; (**d**) cross-correlation functions obtained by GCC with PHAT; (**e**) cross-correlation functions obtained by AGCC.

Figure 8 shows the results of the simulation experiments with $A_{m}=100$ and RE=85.6. Figure 8a shows the original signals and Figure 8b–e are the cross-correlation functions that were obtained by the CC (conventional cross-correlation) method, the GCC method with Roth, the GCC method with PHAT and the new AGCC method, respectively.

In Figure 8a, it is difficult to observe any random fluctuations and the time delay of the two signals with the naked eye. The peak position of the cross-correlation functions that were obtained by CC was τ=−4 ms, as shown in Figure 8b. In Figure 8c,d, it can be seen that the peak at τ=0 ms increased so much that the peak in the correct position almost disappeared. In Figure 8e, it can be observed that the cross-correlation function could still realize effective time delay estimation.

From Table 1 and Figure 7 and Figure 8, it can be seen that when the ratio of the signal energy RE was lower than 10.0, all of the different methods could implement time delay estimation successfully. With the increase in the degree of periodicity, the basic cross-correlation method and the GCC methods with PHAT and Roth all failed in time delay estimation when the RE was higher than 30.0. However, the proposed new AGCC method could work effectively until the RE was higher than 190.0.

**Figure 9:**
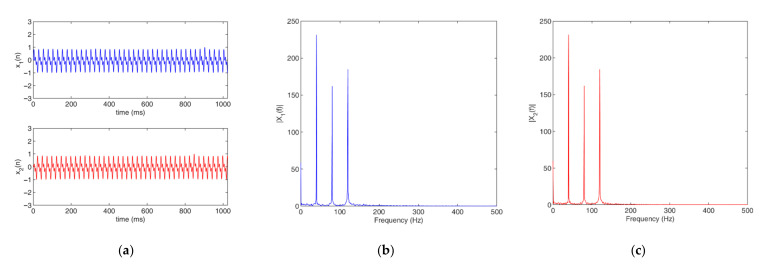
A typical example of signals with three strong periodicity components: (**a**) signals; (**b**) spectra of $x_{1}\left(t\right)$; (**c**) spectra of $x_{2}\left(t\right)$.

Simulation experiments were also carried out to verify the effectiveness of the modified AGCC method for signals with strong periodicity that had multiple strongly periodical components, as proposed in Section 2.3, Figure 6. Figure 9 shows a typical example of signals with strong periodicity that have three strongly periodical components.

**Figure 10:**
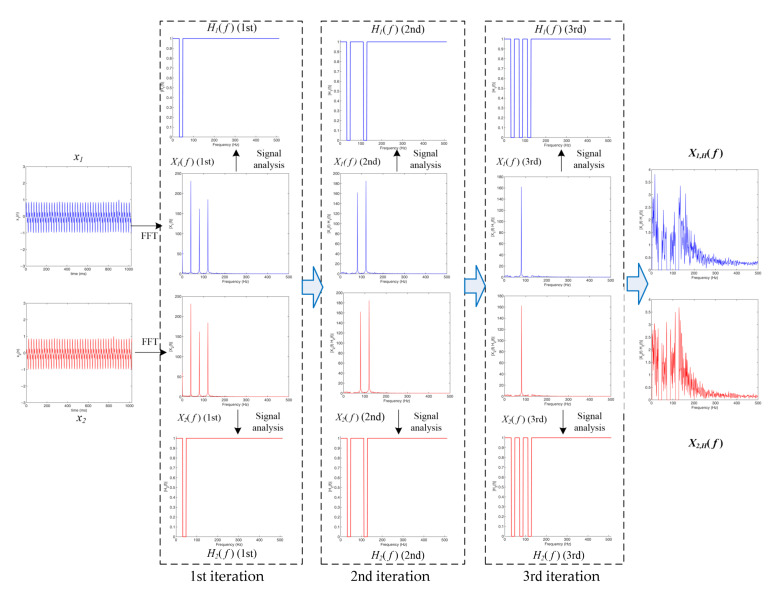
Obtaining the spectra of the filtered signals $X_{1,H}\left(f_{i}\right)$ and $X_{2,H}\left(f_{i}\right)$ for signals with three strong periodicity components.

**Figure 11:**
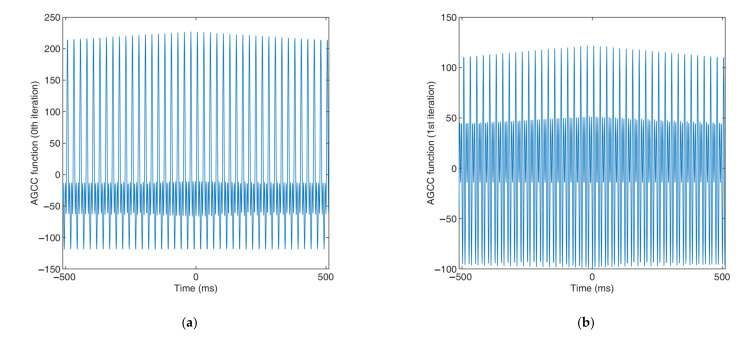

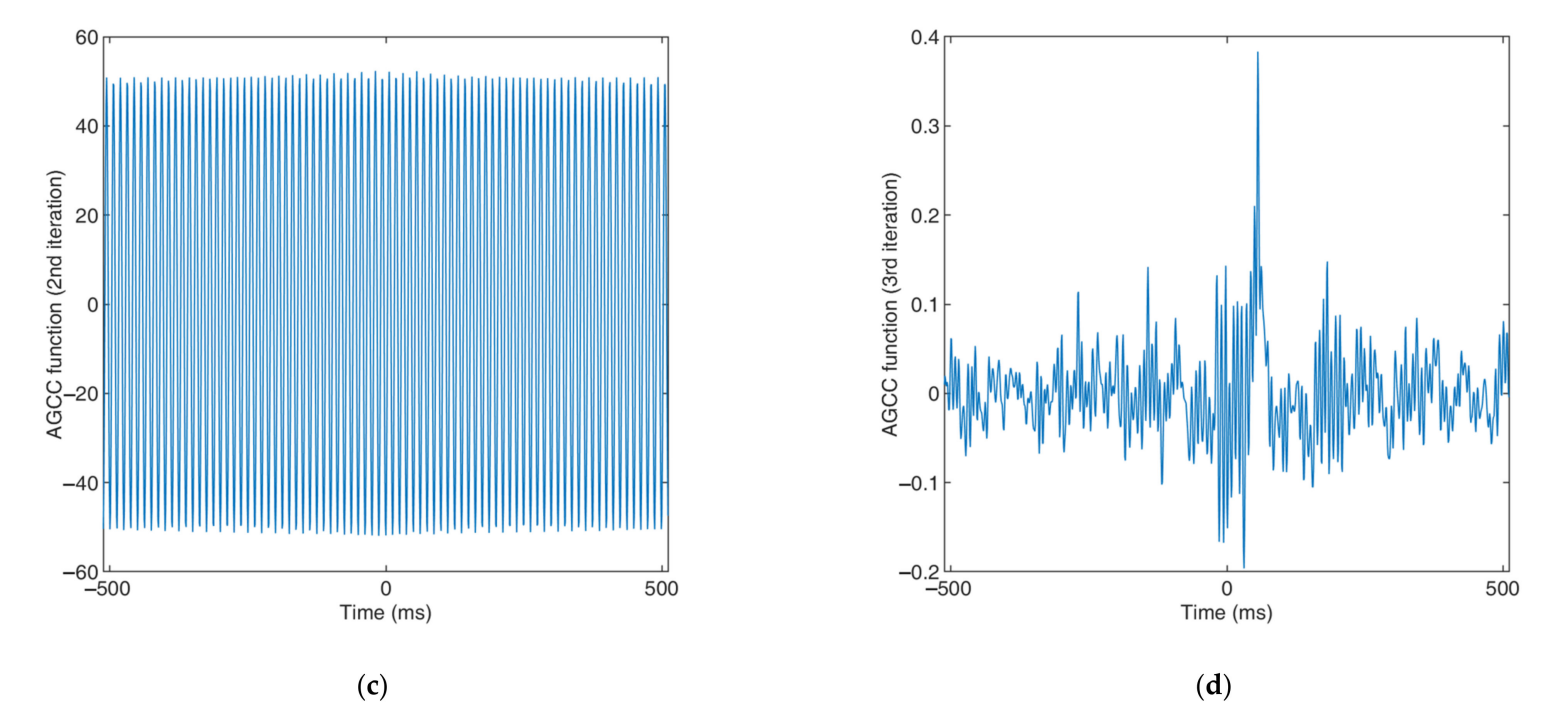
AGCC functions obtained by the signal with different iteration numbers: (**a**) iteration number j=0; (**b**) iteration number j=1; (**c**) iteration number j=2; (**d**) iteration number j=3.

As shown in Figure 9, there were three prominent and obvious peaks in the frequency, which corresponded to the strong periodicity components. Figure 10 shows the process for using the new AGCC method to implement the time delay estimation of signals with strong periodicity that have multiple strongly periodical components (using the signals in Figure 9 as an example). Figure 10 shows the process for obtaining the spectra of the filtered signals $X_{1,H}\left(f_{i}\right)$ and $X_{2,H}\left(f_{i}\right)$ and Figure 11a–d show the cross-correlation functions that were obtained by the signal without filtration (iteration number j=0) and by the signals that were filtered for iteration numbers j=1, j=2 and j=3, respectively.

As shown in Figure 10, a total of three iterations were carried out for the signals with strong periodicity that had three strongly periodical components and each iteration suppressed a strongly periodical component. Figure 11 shows the necessary modification of the AGCC method for this kind of signal and the effectiveness of this modification. By comparing Figure 11d and Figure 11a–c, it is obvious that the AGCC method could only implement effective time delay estimation after three iterations, i.e., when not all strongly periodical components were suppressed, the AGCC method could not work effectively.

### 3.2. Measurement of Slug Flow Velocity in Small Channels

To further verify the effectiveness of the proposed adaptive GCC method, the measurement of slug flow velocity in small channels with inner diameters of 2.0 mm, 2.5 mm and 3.0 mm were carried out.

Velocity is a basic parameter of gas–liquid two-phase flow that plays a role in the quality control, process safety and production efficiency of industrial processing. Velocity is also the basis for the further academic study of gas–liquid two-phase flow. Meanwhile, the new AGCC method that is proposed in this work would be suitable for the measurement of slug flow velocity in small channels.

**Figure 12:**
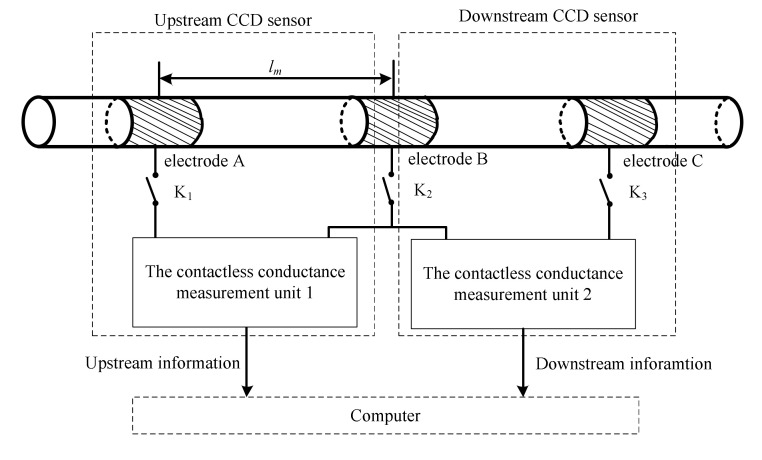
Construction of the velocity measurement system [28].

The velocity measurement system was developed to obtain flow information about the slug flow using a CCD sensor. Figure 12 shows the construction of the velocity measurement system. For more detail information on the CCD and the velocity measurement system, please refer to [28].

**Figure 13:**
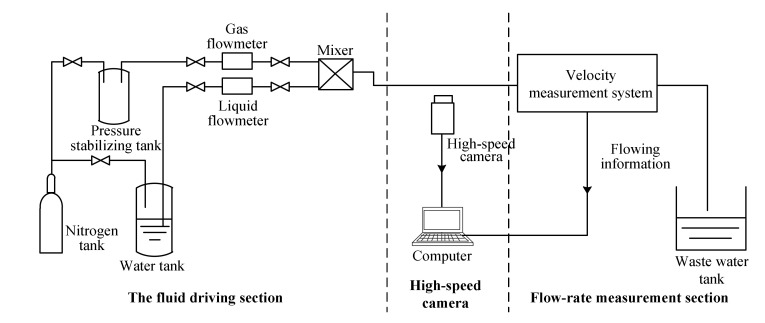
The experimental setup.

Figure 13 shows the experimental setup that was used for the flow rate measurements in this work.

The experimental setup included a fluid driving section, a high-speed camera and the flow rate measurement section. The fluid driving section consisted of a high-pressure nitrogen tank, a water tank, a pressure stabilizing tank, a gas flowmeter, a liquid flowmeter and a mixer. The experimental materials were tap water and nitrogen. The tap water and nitrogen were driven into the small channel by the high-pressure nitrogen. The reference liquid flow rate was measured using an electromagnetic flowmeter (IFC-300C, Krohne, Duisburg, Germany) and the gas flow rate was measured and controlled using a thermal gas flowmeter (F-201 CB, Bronkhorst, Bethlehem, USA). By adjusting the flow rates of the liquid and gas, different slug flows could be obtained and displayed in the experimental channel. An electromagnetic flowmeter was also used to obtain the reference flow rate data. The high-speed camera was used to obtain the reference velocity data.

In the practical velocity measurements, the measured velocity was:

(25) $$v_{slug}=k\frac{l}{\tau }$$

where k is the calibration coefficient, l is the distance between the upstream sensor and the downstream sensor (in this work, l=30.0 mm) and τ is the time delay that was estimated by the proposed AGCC method. The relative errors $e_{v}$ between the measured velocity and the reference velocity were used as the measurement performance index. The definition of $e_{v}$ was:

(26) $$e_{v}=\frac{v_{m}-v_{\mathrm{ref}}}{v_{\mathrm{ref}}}\times 100\%$$

**Figure 14:**
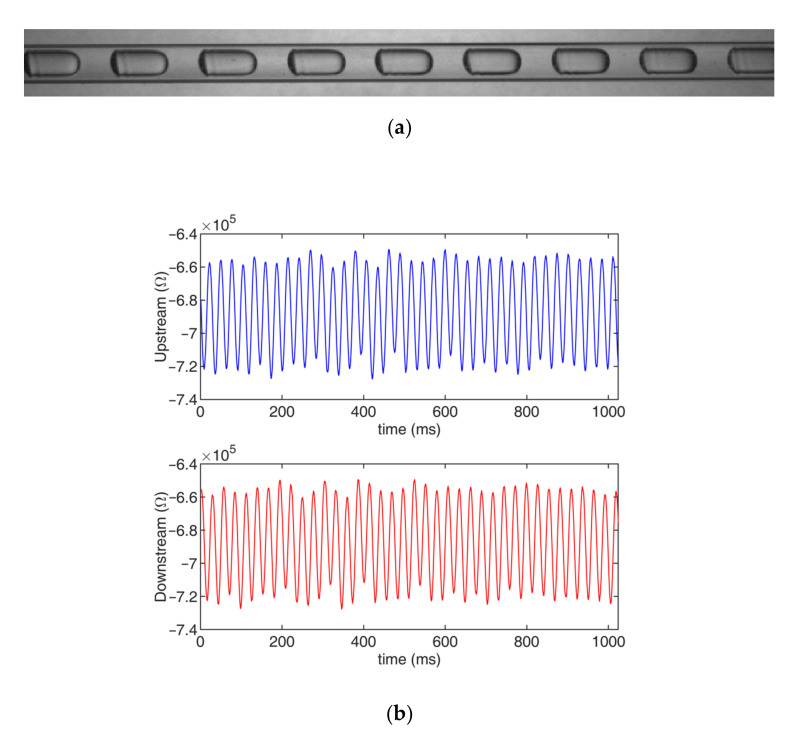
Typical slug flow with strong periodicity (experimental conditions: velocity, 0.41 m/s; time delay, 73.2 ms): (**a**) photo; (**b**) signals.

Figure 14 shows a typical example of a slug flow with strong periodicity that was obtained using the high-speed camera and the upstream and downstream signals from the velocity measurement system.

**Figure 15:**
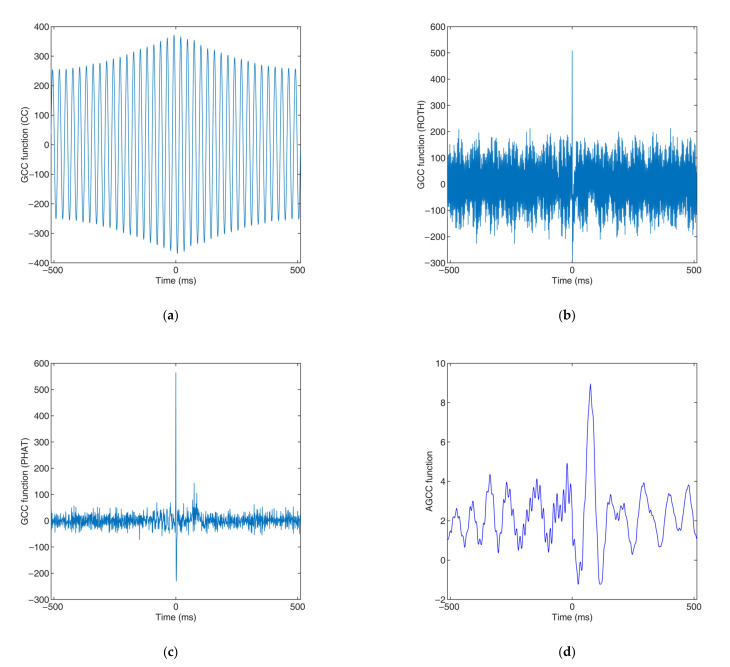
CC and GCC functions of slug flow with strong periodicity: (**a**) basic CC (obtained time delay, −7.0 ms; measured velocity, −4.29 m/s; $e_{v}=-1145.30\%$); (**b**) GCC method with Roth (obtained time delay, 0 ms; no meaningful measured velocity); (**c**) the GCC method with PHAT (obtained time delay, 0 ms; no meaningful measured velocity); (**d**) the proposed AGCC method (obtained time delay, 75 ms; measured velocity, 0.40 m/s; $e_{v}=-2.44\%$).

Figure 15 shows the CC and GCC functions of the slug flow in Figure 11 that were obtained by the basic CC method, the GCC method with Roth, the GCC method with PHAT and the proposed AGCC method.

As shown in Figure 14 and Figure 15, when the slug flow had strong periodicity, the conductance information that was obtained by the CCD sensor consisted of signals with strong periodicity. With the proposed AGCC method, the velocity measurement could be realized successfully, while the other CC and GCC methods could not measure the velocity effectively.

Table 2 lists the velocity measurement results for the three channels.

The experimental results showed that the measurement of slug flow velocity in small channels using the new AGCC method was effective. In the three small channels, the maximum relative errors of the slug flow velocity measurements were all less than 4.50%.

## 4. Conclusions

In this work, a new adaptive generalized cross-correlation (AGCC) method was proposed to implement the time delay estimation of signals with strong periodicity. The new AGCC algorithm analyzes the input signals first and hence, judges whether the input signals have strong periodicity. When the input signals have strong periodicity, the method determines the center frequencies of the strongly periodical components. According to the results of the signal analysis, band-stop filters are designed adaptively to suppress the strongly periodical components. Then, by calculating the mutual power spectral density, obtaining the processed AGCC function and finding the peak of the processed AGCC function, the time delay estimation can be implemented.

Simulation experiments and practical velocity measurement experiments were carried out to verify the effectiveness of the proposed AGCC method. In the simulation experiments, the time delay estimation of test signals with different degrees of periodicity was implemented using the basic cross-correlation method, the GCC methods with Roth and PHAT and the proposed AGCC method. The experimental results showed that with the increase in the degree of periodicity, the basic cross-correlation method and the GCC methods with Roth and PHAT all failed in the time delay estimation and that the time delay estimation results had large errors. However, the proposed new method could still work effectively.

The practical velocity measurement experiments were carried out using a CCD sensor and the new AGCC method. The new AGCC method was adapted to process the obtained upstream signal and downstream signal and hence, realized the measurement of slug flow velocity in small channels. The practical velocity measurement experiments were carried out in small channels with inner diameters of 2.0 mm, 2.5 mm and 3.0 mm in order to verify the effectiveness of the new AGCC algorithm and the new velocity measurement method. The experimental results showed that the measurement performance of the proposed method was satisfactory. The problems that are associated with using the normal CC method for the measurement of slug flows with strong periodicity can be overcome and the maximum errors were less than 4.50%.

Both the simulation experiments and the practical velocity measurement experiments indicated that the proposed AGCC method could implement the effective time delay estimation of signals with strong periodicity and that the problems in time delay estimation that are caused by signals with strong periodicity, such as the cross-correlation function not having an obvious peak and the maximum position of the cross-correlation function not representing the practical time delay, can be overcome. Furthermore, the successful application of the proposed AGCC method for slug flow velocity measurement also showed the potential of the proposed method within the research field of parameter measurement.

In practical applications, the proposed AGCC method could realize the effective time delay estimation of signals with strong periodicity and the band-stop filters could suppress the strongly periodical components. However, the existing method requires a relatively large amount of computation and some a priori knowledge to be introduced. Our future research work will aim to develop faster and smarter signal processing methods (such as the Hilbert transform) and combine them with the GCC method to overcome the unfavorable influences of the strong periodicity of signals.

## Figures and Tables

**Table 1:** Experimental results of the simulation experiments.

$A_{m}$	RE	Reference Time Delay (ms)	Time Delay Estimation Result (ms)			
			CC	GCC (Roth)	GCC (PHAT)	Proposed AGCC
1	0.01	56	56	56	56	56
10	0.9	56	56	56	56	56
20	3.5	56	56	56	56	56
30	7.6	56	56	56	56	56
40	13.5	56	56	56	0	56
50	21.2	56	−4	56	0	56
60	30.5	56	−4	0	0	56
70	41.5	56	−4	0	0	56
80	51.1	56	−4	0	0	56
90	68.5	56	−4	0	0	56
100	85.6	56	−4	0	0	56
110	102.3	56	−4	0	0	56
120	121.8	56	−4	0	0	56
130	142.0	56	−4	0	0	56
140	165.8	56	−4	0	0	56
150	190.5	56	−4	0	0	56

Note: In the simulation experiments, the length of the test signal was 1000, $f_{c}=50\;\mathrm{Hz}$ and Δt=1.0 ms. The simulation experiments were carried out using MATLAB.

**Table 2:** Velocity measurement results.

Inner Diameter (mm)	Velocity Range (m/s)	$e_{v}$
2.0	0.52~1.32	−3.52~4.31%
2.5	0.22~1.15	−3.91~4.23%
3.0	0.52~1.09	−4.16~3.58%

## Data Availability

The data presented in this study are available on request from the corresponding author. The data are not publicly available due to policy reasons.
